# Electrocardiography as the First Step for the Further Examination of Cardiac Involvement in Myasthenia Gravis

**DOI:** 10.1155/2016/8058946

**Published:** 2016-01-17

**Authors:** Takao Kato, Sayako Hirose, Shogo Kumagai, Akihiko Ozaki, Sadayuki Matsumoto, Moriaki Inoko

**Affiliations:** ^1^Cardiovascular Center, Tazuke Kofukai Medical Research Institute, Kitano Hospital, Osaka 530-8480, Japan; ^2^Department of Cardiovascular Medicine, Graduate School of Medicine, Kyoto University, Kyoto 606-8507, Japan; ^3^Department of Neurology, Tazuke Kofukai Medical Research Institute, Kitano Hospital, Osaka 530-8480, Japan

## Abstract

*Introduction*. Cardiac involvement of myasthenia gravis (MG) accompanies a poor prognosis. In the present study, we aimed to investigate the relationship between ECG abnormality and cardiac involvement.* Methods*. Of 178 patients diagnosed with MG between 2001 and 2013 at our hospital, we retrospectively analyzed consecutive 58 patients who underwent both ECG and echocardiography and without underlying cardiovascular disease. ECG abnormalities were defined by computer-assigned Minnesota-codes. Cardiac damage was defined as either (1) ejection fraction (EF) <55% on echocardiography or (2) elevated *E*/*e*′, the ratio of mitral velocity to early diastolic velocity of the mitral annulus >8 on echocardiography.* Results*. Thirty-three patients (56.8%) had ECG abnormality. An elevated *E*/*e*′ was observed in patients with ECG abnormality compared to those without ECG abnormality (11.2 ± 3.2, 8.7 ± 2.2, resp., *p* = 0.03). Among patients with ECG abnormality, 14 of 15 patients showed cardiac damage. Among patients without ECG abnormality, 6 of 33 patients showed cardiac damage (*p* = 0.003). Reduced EF was observed in five patients (8.6%) with ECG abnormality and none in patients without ECG abnormality.* Conclusions*. ECG may aid as the first step for the further examination of cardiac damage in patients with MG.

## 1. Introduction

Myasthenia gravis (MG) primarily affects skeletal muscle via production of autoantibodies against nicotinic acetylcholine receptors in the motor endplates; heart muscle is a rare autoimmune target [1, 2]. Patients with MG may show several kinds of cardiac involvement, ranging from asymptomatic changes on electrocardiography (ECG) to ventricular tachycardia, myocarditis, conduction disorder, heart failure, and sudden death [2–5]. Detection of cardiac involvement in MG is important because this cardiomyopathy may be treatable [4, 5]. In the present study, we aimed to investigate the relationship between ECG abnormality and cardiac damage by echocardiography in order to aid in early detection and timely treatment.

## 2. Methods

Of 178 patients diagnosed with MG between 2001 and 2013 at our hospital, we retrospectively analyzed consecutive 58 patients who underwent both ECG and echocardiographic examinations and without underlying cardiovascular disease (ischemic heart disease, valvular heart disease, and hypertensive heart disease). ECG abnormalities were defined by standardized computer-assigned Minnesota-codes [6, 7]. Echocardiography was performed by cardiac sonographers according to the guidelines [8].

Cardiac damage was defined as either (1) ejection fraction (EF) <55% on echocardiography (the EF of a normal heart may be between 55% and 70%) according to the practice guideline [8, 9] or (2) elevated *E*/*e*′, the ratio of mitral velocity to early diastolic velocity of the mitral annulus (*E*/*e*′) >8 on echocardiography (the *E*/*e*′ of a normal left ventricular end-diastolic pressure may be <8) [8, 10].

The study conformed to the ethical guidelines of the 1975 Declaration of Helsinki and was approved by the Institutional Review Board of Kitano Hospital. All data are expressed as mean ± SD. Differences between groups were compared by using the Mann-Whitney *U* test, while categorical variables were compared by using Pearson's chi-square test. In all tests, *p* values of <0.05 were considered significant.

## 3. Results

Thirty-three patients (56.8%) had ECG abnormality: 10.3% had atrial fibrillation, 6.8% had atrioventricular block, 29.3% had nonspecific ST depression, and 29.3% had negative T wave (Table 1). There were no differences in comorbidities or clinical characteristics of MG patients with or without ECG abnormality (Table 2). On echocardiography, there was a difference in *E*/*e*′ between patients with or without ECG abnormality (11.2 ± 3.2, 8.7 ± 2.2, resp., *p* = 0.03, Table 3). Among patients with ECG abnormality, 14 of 15 patients showed the suggested cardiac damage. Among patients without ECG abnormality, 6 of 33 patients showed the suggested cardiac damage (*p* = 0.003, Table 4). The sensitivity of ECG abnormality for detecting cardiac damage was 0.700 (95% confidence interval [CI], 0.525–0.836), while its specificity was 0.711 (95% CI, 0.618–0.782). The positive predictive value was 0.560 (95% CI, 0.420–0.669), while the negative predictive value was 0.818 (95% CI, 0.712–0.901).

When we limited the cases by reduced EF, five patients (8.6%) had reduced EF (EFs of 10%, 26%, 43%, 49%, and 54%, resp.) with diffuse hypokinesis; all 5 had ECG abnormality with high *E*/*e*′ values of 12 ± 3.6. Among patients without ECG abnormality, none of the patients showed reduced EF. Of the 5 patients with reduced EF, 1 patient with an EF of 30% died of colon cancer, and 4 patients were administered immunosuppressive therapy. Three of the 4 patients with EFs of 43%, 47%, and 54%, respectively, had a good clinical course, while 1 patient with an EF of 10% died of acute decompensated heart failure approximately 1 month after self-discontinuation of immunosuppressive therapy.

## 4. Discussion

Cardiac manifestation is thought to be relatively rare in MG. However, it has been reported that approximately 16% of patients with MG have various cardiac manifestations, such as arrhythmia and heart failure [1]. Autoimmunologic mechanisms are considered to be contributory to such cardiac involvement [2], such as the antibody for ryanodine receptor (RyR), the antibody for both beta 1 and beta 2 adrenergic receptors, the anti-titin antibodies, and the antibody for Kv1.4—a muscular voltage-gated potassium channel [2]. Arrhythmia is the most common clinical manifestation of unexplained cardiac disease in MG, occurring in as many as 8% of cases [1], which is consistent with the findings of the present study. The prevalence of abnormal ECG is also reported varying from 16% to 88% of patients with MG [1, 11, 12], probably due to the inclusivity of MG patients who underwent ECG; however, no demonstration of any clinical significance was provided. This study validated the presence of ECG abnormality in patients with MG, including arrhythmia, conduction abnormality, and the abnormality of ST portion and T wave. We also showed that a small number of patients without ECG abnormality demonstrated cardiac damage, which comprised only diastolic impairment and did not include systolic impairment. These results implied the utility of ECG. If the abnormality exists, further examination by echocardiography may be necessary for the detection of systolic impairment in patients with MG, which indicates a poor prognosis but can be treated by immunosuppressant therapy [1, 2, 4]. Therefore, the presence or absence of ECG abnormality may be useful as the first step for the further examination of cardiac damage in patients with MG.

Cardiac investigation in patients with MG with unexplained fatigue should comprise a physical examination including third tone, holosystolic murmurs, ankle swelling, or lung rales; laboratory findings including brain natriuretic peptide (BNP) [8–10] and cardiac troponin [4]; 6-minute walk distance [13]; and echocardiography along with ECG. Diastolic impairment also affects the exercise intolerance and it is more likely to cause atrial fibrillation. *E*/*e*′ in particular closely correlates with left ventricular filling pressure. Several prominent validation studies have confirmed this correlation and noted that the prediction of normal or abnormal filling pressure is most reliable when the ratio is <8 or >15, respectively [14, 15]. In our study, the *E*/*e*′ values were relatively high (mean: 10.46 ± 3.24) in patients in MG. There is a possibility that diastolic function was reduced in patients with MG. The diameter of the left atrium tends to be larger in patients with ECG abnormality; however, the combined measurement of BNP or N-terminal pro-BNP levels requires validation [8–10]. The mechanism underlying the high *E*/*e*′ could be explained, at least partly, by the disturbance of calcium handling related to ryanodine receptors [16], which are one of the targets of autoimmunity in patients with MG [1]. A large-scale, long-term study is needed for verification, while the mechanism and clinical significance of diastolic dysfunction also remain to be elucidated.

There are several limitations of the present study. First, not all the patients with MG underwent ECG and echocardiographic examinations at our hospital and a selection bias may exist. Therefore, further prospective large-scale study or the analysis of consecutive cases is warranted to validate ECG as a useful tool for the screening method of cardiac dysfunction in all patients with MG. Second, coronary artery disease could not be excluded entirely in these patients with MG, even though regional abnormality of wall motion, consistent with coronary artery disease, was not found in the present study. Third, whether an exercise test provoked the reduction of EF was not verified in the present study. BNP (or N-terminal pro-BNP) levels and physical findings were inadequate to be acquired. However, due to the rarity of the MG with cardiac involvement, this retrospective study has a clinical insight for the detection of systolic or diastolic dysfunction in patients with MG.

## 5. Conclusions

ECG may aid as the first step for the further examination of cardiac damage in patients with MG.

## Figures and Tables

**Table 1:** Characteristics of electrocardiographic abnormality.

Characteristic	*n* (%)	With left ventricle damage, *n*
Persistent atrial fibrillation	6 (10.3)	3
Atrioventricular block	4 (6.8)	3
ST depression	17 (29.3)	9
Negative T wave	17 (29.3)	8

**Table 2:** Clinical characteristics of patients with myasthenia gravis.

	With ECG abnormality (*n* = 33)	Without ECG abnormality (*n* = 25)	*p* value
Male, *n* (%)	15 (48.4)	9 (50.6)	0.903
Age, mean ± SD, y	60 ± 5.2	58 ± 6.1	0.67
Time from diagnosis, mean ± SD, y	12 ± 8.0	11 ± 7.1	0.67
Thymoma, *n* (%)	8 (24.2)	10 (40)	0.08
Comorbidities, *n*			
Diabetes mellitus	1	2	
Systemic lupus	1	2	
Rheumatoid arthritis	0	1	
Sjogren disease	0	1	
Death, *n*	3	3	0.512
Cause of death, *n*			
Malignancy	1	2	
Heart failure	1		
Acute myocardial infarction	1		
Aplastic anemia		1	
Type of treatment, *n* (%)			
Steroid	19 (55.4)	10 (40.0)	
Cholinesterase inhibitor	14 (34.5)	7 (28.0)	
Immunosuppressant	12 (34.2)	11 (44.0)	

ECG: electrocardiography; LV: left ventricle.

**Table 3:** Echocardiographic parameters of patients with myasthenia gravis.

	With ECG abnormality (*n* = 33)	Without ECG abnormality (*n* = 25)	*p* value
Ejection fraction (%)	61.2 ± 15.1	66.1 ± 4.8	0.16
LV end-diastolic dimension (mm)	44.7 ± 5.8	47.6 ± 7.3	0.14
LV end-systolic dimension (mm)	31.8 ± 9.5	28.4 ± 4.3	0.90
Septal wall thickness (mm)	8.9 ± 1.6	8.8 ± 1.1	0.63
*E*/*e*′	11.2 ± 3.2	8.7 ± 2.2	0.03
Left atrium diameter (mm)	36.5 ± 6.6	34.1 ± 5.6	0.20
Wall motion abnormality	5; diffuse	0	

LV: left ventricle.

**Table 4:** Data concerning the LV damage and ECG abnormality.

	With LV damage	Without LV damage	(*n*)
With ECG abnormality	14 (5 with reduced EF)	11	25
Without ECG abnormality	6 (0 with reduced EF)	27	33
(*n*)	20	38	58
